# Vaginal Microflora in High Vaginal Swab in Prelabour Rupture of Membrane: A Descriptive Cross-sectional Study

**DOI:** 10.31729/jnma.8737

**Published:** 2024-08-31

**Authors:** Jyotshna Sharma, Sanjeeb Tiwari, Durga Thapa, Ranjana Yadav

**Affiliations:** 1 Department of Obstetrics and Gynaecology, Kathmandu Medical College and Teaching Hospital, Sinamangal, Kathmandu, Nepal; 2 Department of General Practice and Emergency Medicine, Tribhuvan University Teaching Hospital, Maharajgunj, Kathmandu, Nepal

**Keywords:** *escherichia coli*, *premature rupture of membrane*, *preterm birth*

## Abstract

**Introduction:**

Premature rupture of membrane (PROM) refers to the disruption of the fetal membrane before the beginning of labor, resulting in spontaneous leakage of amniotic fluid. Cervicovaginal infection is an important risk factor of PROM and can lead to complications to mother and the child. This study aimed to delineate the bacterial patterns found in PROM so that the ideal appropriate responsive antibiotics can be chosen.

**Methods:**

A descriptive cross-sectional was done during the period of 6 months from December of 2023 to May 2023, to characterize the microorganisms in the vaginal fluid found in antenatal women presenting with premature rupture of membraneafter obtaining ethical approval (IRC number: 20102023/02). A total of 117 antenatal women diagnosed with premature rupture of membrane were included in the study. High vaginal swabs were collected for microbial culture and sensitivity. Data were entered using Microsoft Excel 2000 (v9.0) and Statistical Package for the Social Sciences (SPSS) software version 26.0 was used for analysis.

**Results:**

Out of 117 samples, culture growth was present in the culture of high vaginal swabs of 23 (19.66%) women. The high vaginal swab cultures from the samples collected in women presenting with PROM reported 9 different types of pathogens *E. coli* in 12 (52.17%), *Klebsiella* in 4 (17.39%) and *Pseudomonas* in 2 (8.70%) cultures.

**Conclusions:**

This study reveals diverse microorganisms in premature rupture of membrane cases, with E. coli being the most common. Identifying these bacterial patterns is essential for selecting effective antibiotics, improving maternal and neonatal outcomes, and reducing morbidity and mortality by early detection and treatment of vaginal infections.

## INTRODUCTION

Premature rupture of membrane (PROM) refers to the disruption of fetal membrane before the beginning of labor, resulting in spontaneous leakage of amniotic fluid.^1^ Preterm premature membrane rupture occurs in about 1% of all pregnancies and accounts for 30-40% of preterm births.^2^ The cause of PROM is multifaceted, with cervicovaginal infections being the most significant.^3^ The other specific risk factors for PROM were BMI <18.5 kg/m^2^, history of PROM, nulliparity, and low level of education.^4^

Studies show that women with both intrauterine infection and preterm PROM are more likely to experience abruption compared to those without these complications and there is improvement in neonatal and maternal outcomes when antibiotics are used in PROM.^5,6^ Thus, understanding the bacterial patterns specific to PROM can guide the selection of appropriate antibiotics, thereby potentially improving maternal and neonatal outcomes.

This study aims to find the bacterial patterns found in PROM so that the ideal appropriate responsive antibiotics can be chosen.

## METHODS

It is a descriptive cross-sectional study that was done at a tertiary care center in the Department of Obstetrics and Gynecology, Kathmandu Medical College and Teaching Hospital,Kathmandu, Nepal. This study was conducted for 6 months after getting ethical clearance from the Institutional Review Committee of the institution (IRC number: 20102023/02) on 23^rd^ October 2023. All Pregnant women with a diagnosis of PROM established after proper history and examination after 28 weeks of gestation presenting to the OPD or labor room were enrolled for the study. The data was collected from December of 2023 to May 2023. Exclusion criteria were antenatal women presenting with gestational age less than 28 weeks, patients with feature of chorioamnionitis like fever, tachycardia, uterine tenderness or foul smelling liquor, fetal distress and meconium stained amniotic fluid on admission, patients having active labor on admission, patients presenting with antepartum hemorrhage. Total sampling method was used and all the cases of PROM during the study period of six months were taken which were 117 in number.

Informed permission was obtained from the patients before the data collection. When the patient arrived at the hospital, a comprehensive history, physical examination, and obstetrical examination were performed. By doing a sterile speculum examination, observing a pool of amniotic fluid in the posterior fornix of the vagina, and noting the fluid exiting the body through the cervical os with or without the Valsalva maneuver, the diagnosis of PROM was established. Using the sterile swab sticks that are sold commercially, high vaginal swabs were obtained while adhering to all aseptic procedures during perspeculum examination. The swabs were sent for cultures in the lab. After the culture report was obtained data were entered using Microsoft Excel 2000 (v9.0) and Statistical Package for the Social Sciences (SPSS) software version 26.0 was used for analysis.

## RESULTS

A total of 117 pregnant women with PROM were included in this study. Culture growth was present in the culture of high vaginal swabs of 23 (19.66%) women and was absent in the remaining 94 (80.34%) women. The high vaginal swab cultures from the samples collected in women presenting with PROM reported 9 different types of pathogens. The organisms detected were E. coli in 12 (52.17%), Klebsiella in 4 (17.39%) and Pseudomonas in 2 (8.70%) cultures. The age groups of participants in this study is between 18-40 years with the mean age being 28.66±4.76 years. The women with PROM in the age range of 20-34 years were 101 (86.32%) (Table 1).

Among 117 women, 66 (56.41%) were primigravida , 64 (54.70%) women had normal vaginal delivery. The percentage of PROM cases were seen after 37 weeks of gestation was 72 (61.54%) and 45 (38.46%) cases occurred before 37 weeks of gestation (Table 2).

**Table 1:** Age distribution of Women (n=117)

Age (years)	n (%)
<19.99	4 (3.42)
20-34	101 (86.32)
≥35	12 (10.26)

**Table 2:** Obstetric information of Women (n=117)

Parity	n (%)
Primigravida	66 (56.41)
Multigravida	51 (43.59)
**Mode of Delivery**	
Normal Vaginal Delivery	64 (54.70)
Cesarean section	52 (44.44)
Induced Delivery	1 (0.85)
**Period of gestation**	
Term	72 (61.54)
Preterm	45 (38.46)

**Table 3:** Culture growth pattern (n=117)

Culture Growth	n (%)
No	94 (80.34)
Yes	23 (19.66)
*E. Coli*	12 (52.17)
*Klebsiella*	4 (17.39)
*Pseudomonas*	2 (8.70)
*Staphylococci*	1 (4.35)
*Enterobacter*	1 (4.35)
*K pneumoniae And E. Coli*	1 (4.35)
*P. aeruginosa*	1 (4.35)
*Pseudomonas aeruginosa*	1 (4.35)

In our study, for primipara, the presence of culturegrowth is found in 13 (19.69%), of cases of PROM andfor multipara, it is 10 (19.60%)(Table 4).

**Table 4:** Culture growth according to parity (n=117)

Parity	Growth present n(%)	Growth absent n (%)	Total
Primipara	13 (19.69)	53 (80.30)	66 (100)
Multipara	10 (19.60)	41 (80.39)	51 (100)

Culture growth is present in 17 (37.77%) of total preterm pregnancies and 6 (8.33%) of total term pregnancies. Out of 45 preterm pregnancies, 28 (62.22%) did not show culture growth (Table 5).

**Table 5:** Culture growth according to period of gestation (n=117)

Period of gestation	Growth present n(%)	Growth absent n (%)	Total
Term	6 (8.33)	66 (91.67)	72 (100)
Preterm	17 (37.77)	28 (62.22)	45 (100)

## DISCUSSION

In this descriptive cross sectional study we performed culture of high vaginal swab in women with PROM. Escherichia coli was the most common isolated organism followed by Klebsiella and Pseudomonas. In a study conducted by Rani et al. (2014), E. coli was the most common organism associated with PROM similar to our study.^7^ This might have been due to similar sociocultural environment in Nepal and India. A study done by McDonald et al. demonstrated a significant association between E. coli, Klebsiella spp. and PROM.^8^ A study done in India by Ambalpady et al. in 2022 reported culture growth in 85.09% of samples and no growth in remaining 14.91% samples.^9^ This is inconsistent with our study where culture growth is present in 22 (19.66%) of the samples. This might be due to the fact that our study site is located in the urban area where more patients are educated and maintain regular vaginal hygiene and have routine antenatal checkup.

According to Surekha S. Mohan et al (2017), in a study of 358 pregnant women with PROM in India, 61.1% of the patients were multigravida whereas 38.9% were primigravida.^10^ This is inconsistent with our study where the percentage of primigravida is more than multigravida female. The reason behind this inconsistency may have been because it was conducted in a different geographical area, healthcare setting, or with different population characteristics. In a study done in Uganda of 87 pregnant women with PROM, 84% of the women belonged to the age group of 20-34.99 years.^11^ This is comparable with our study where 101 (86.32%) women belong to the age group of 20-35 years. The similarity in the age distribution between suggests that the age range of 20-35 years is a common demographic for women experiencing PROM. This could be due to the fact that this age range encompasses the majority of childbearing years. In our study, the percentages of culture growth presence and absence are quite similar between primipara and multipara. For primipara, the presence of culture growth is 13 (19.69%), and for multipara, it is 10 (19.60%) (Table 4). As both groups have nearly identical percentages of culture growth presence and absence, parity does not seem to have significantly influenced the presence or absence of culture growth in this sample.

According to a study done by Telayneh et al., 32.7% of PROM patients had a gestational age of less than 37 weeks, while 67.3% of cases had a gestational age of more than 37 weeks.^12^ This is similar to our study where 72 (61.54%). PROM cases were seen after 37 weeks of gestation and 45 (38.46%) cases occurred before 37 weeks of gestation. Here, the percentages from both studies are reasonably close, indicating a consistent pattern across different populations. If PROM develops in the early stages of pregnancy, the woman and the unborn child are at great risk. If an infection is found to be the cause, managing the condition becomes more challenging. Our study found a notable difference in the of culture growth pattern between term and preterm pregnancies.

Culture growth is significantly more prevalent in preterm pregnancies 17 (37.77%) compared to term pregnancies 6 (8.33%). The majority of term pregnancies did not show culture growth 66 (91.67%), whereas a lower percentage of preterm pregnancies did not show culture growth 28 (62.22%). It is estimated that 15-25% of women with preterm PROM get postpartum infection, and 15-35% of cases involve clinically apparent intraamniotic infection. At early gestational ages, the incidence of infection is greater.^13^ Reducing the negative consequences of PPROM requires early identification, admission, and initiation of antibiotic treatment.^14^ Studies suggest that antibiotics can lengthen pregnancy and reduce morbidity in the mother and newborn when given to women who are not in labor and there is more evidence of a benefit at early gestational ages (less than 32 weeks).^15^ At these stages, the fetus is particularly vulnerable to complications related to prematurity. Prolonging pregnancy even by a few days can lead to significant improvements in neonatal outcomes. Therefore, timely diagnosis and treatment is very important.

There are certain limitations in our study. Being a single-center based study, the study results cannot be generalized to a broader population. The study included a relatively small sample size of 117 pregnant women, which may not be sufficient to capture the full spectrum of bacterial patterns associated with PROM. Factors such as nutritional status, access to healthcare, and other socio-economic determinants were not assessed, which could have an impact on the prevalence and types of bacterial infections observed in the study.

## CONCLUSIONS

This study highlights the varied diversity of microorganisms in cases of premature rupture of membranes (PROM), with E. coli being the most commonly isolated pathogen. The majority of PROM cases occurred in women aged 20-34 years, with a nearly equal distribution between primigravida and multigravida women. Notably, culture growth was more prevalent in preterm pregnancies compared to term pregnancies. Identifying the bacterial patterns associated with PROM is crucial for selecting appropriate antibiotic treatments, which can improve maternal and neonatal outcomes. Patients with risk need to be detected early and correct treatment of vaginal infection can reduce the chances of morbidity and mortality.
